# The Influence of Hot-Dip Galvanizing on the Mechanical Properties of High-Strength Steels

**DOI:** 10.3390/ma14185219

**Published:** 2021-09-10

**Authors:** Milan Šmak, Jaroslav Kubíček, Jiří Kala, Kamil Podaný, Jan Vaněrek

**Affiliations:** 1 Faculty of Civil Engineering, Brno University of Technology, 601 90 Brno, Czech Republic; kala.j@fce.vutbr.cz (J.K.); vanerek.j@fce.vutbr.cz (J.V.); 2 Faculty of Mechanical Engineering, Brno University of Technology, 616 69 Brno, Czech Republic; kubicek@fme.vutbr.cz (J.K.); podany@fme.vutbr.cz (K.P.)

**Keywords:** hot-dip galvanizing, zinc coating, high-strength steels, yield strength, tensile strength, hardness, thermal process

## Abstract

Modern high-strength steels achieve their strength exclusively through the manufacturing process, as the chemical composition of these steels is very similar to the composition of standard-quality steels. Typically, hot-dip galvanizing is used to form a protective zinc layer on the steel parts of structures; nonetheless, the material is exposed to high temperatures during the process. With high-strength steels, this can lead to deterioration of the mechanical properties. This study aims to experimentally examine and evaluate the extent of deterioration of the mechanical properties of high-strength-steel members. The effect was studied on specimens made of three different types of steel with the yield strength ranging from 460 to 1250 MPa. For each type of steel, selected mechanical properties—yield strength, tensile strength, and hardness—were determined on specimens with and without hot-dip galvanization, and the obtained results were mutually compared. Our study shows a significant impact of the hot-dip galvanization process on the mechanical properties of some high-strength steels. With the studied types of steel, the yield strength decreased by up to 18%, the tensile strength by up to 13%, and the hardness by up to 55%.

## 1. Introduction

Steels are commonly classified as high-strength steels if their yield strength exceeds 460 MPa. Traditionally, high-strength steels are employed in engineering structures and mechanical engineering products. In the construction industry, high-strength steels can be used for either entire load-bearing structures, or key (high-stressed) structural components only. With this advantage, they are used in structures such as silos, tanks, hoppers, towers, and masts; for load-bearing structures in manufacturing facilities; and for load-bearing elements in bridges and footbridges [1]. These applications are implied by their high strength, which allows the transmission of high-intensity loads while using more economically sized elements [2].

The structures are typically exposed to conditions causing atmospheric corrosion. Atmospheric corrosion of structures and structural members made from steel represents an important technical and economic problem, which concerns standard-quality steels (i.e., steels with a yield strength of up to 460 MPa, mainly steels S235 and S355) as well as high-strength steels. Hence, the protection of the surface of steel members constitutes an important issue. Hot-dip galvanizing is one of the most common ways of protecting steel elements against corrosion due to the cost-effectivity and low impact of the process on the environment. Its main advantages include long-term corrosion protection (long-term experience shows that it can last up to 50 years [3]), a high level of mechanical resilience, and perfect all-round protection in cavities and on edges. The lifetime of zinc coating is inversely proportional to the corrosion rate. Table 1 shows that depending on the category of corrosion aggressivity of the atmosphere (given by EN ISO 9223 [4]), the protection of steel members by zinc coating can be very effective.

The chemical composition of steels with a high level of yield strengths of up to 700 MPa is very similar to the composition of standard-quality structural steels, and modern high-strength steels achieve their strength exclusively by way of the manufacturing process [5]. Within the process, the steels are subjected to controlled thermomechanical rolling, rolling with quenching (accelerated cooling is provided by intensive jets of cold water), and subsequent tempering. This sophisticated process reduces the requirements on the content of alloying elements. Quenched and tempered high-strength steels have low values of carbon equivalent, which improves their weldability.

The process of hot-dip galvanizing involves dipping a steel element into a kettle with molten zinc. The temperature of molten zinc is, however, approximately 450 °C [6]. Zinc and steel react under these conditions with one another, forming iron–zinc alloy layers on the surface of the steel [7].

With standard-quality steels, hot-dip galvanizing is a very common and time-proven way of protecting steel structures against the action of atmospheric corrosion. However, the application of hot-dip galvanizing to structures made of high-strength steels can lead to drawbacks related to the loss of strength of the steel. This effect is a consequence of the heat introduced during the hot-dip galvanization process. With certain types of high-strength steels, the manufacturers do not recommend any thermal processing of the steels, as it may lead to deterioration of the mechanical properties (yield strength, tensile strength, and hardness) [8,9,10]. Nonetheless, the extent of the decrease in strength or hardness resulting from the hot-dip galvanizing process is not known for a combination of the different steel types for structural details. The desired mechanical properties may be achieved despite the use of hot-dip galvanizing by selecting suitable high-strength steel while benefiting from complex corrosion protection.

Nowadays, the design of steel structural elements made of both standard-quality steels (S235 to S355) as well as the higher-strength steels S420 and S460, is covered in European standard document EN 1993-1-1 (2006) [11]. Document EN 1993-1-8 (2006) [12] applies to the design of joints and connections, particularly for statically loaded structures made from steel of grade S235 to S460. Last but not least, standard EN 1993-1-12 (2008) [13] supplies additional rules for the use of steels of strength grades up to S700 in structures. Nonetheless, the use of steels with strengths above S700 is not supported in today’s European standard documents. European standard documents also apply to the zinc coatings of high-strength steels. Hot-dip galvanization is a relatively simple process to specify and is covered by the standard EN ISO 1461 [14].

Over the last few decades, companies, public institutions, and organs of state administration have been showing an increasing interest in the influence of products and services on the environment. A study of EGGA/IZA-Europe (European General Galvanizers Association/The International Zinc Association) suggested including hot-dip galvanizing among technologies considered “green,” as it effectively saves natural resources by the efficient protection of steel [15]. Hence, it lowers the energy requirements in comparison to standard coatings.

Recently, environmentally clean technologies have been requested. These include, for example, laser technologies [16,17]. The coating applied by these technologies shows good adhesion to the steel surface, good corrosion protection, and mechanical resistance. However, the technologies are more suitable for small products than civil engineering structures or large machine structures. An alternative technology for corrosion protection is thermal spray methods, employing flame, plasma, or an electric arc. Zinc and aluminum alloys (e.g., Zinacor 850) are mainly used for spraying.

A more precise specification of the negative influence of hot-dip galvanizing on the mechanical properties of high-strength steels is not provided in the standard documents. This paper deals with the issue, and the extent of the negative influence of hot-dip galvanizing on the mechanical properties (strength and hardness) is discussed in detail. The main focus is the experimental assessment of the influence of hot-dip galvanizing on the mechanical properties of selected high-strength steels with yield strength ranging from 460 to 1250 MPa.

## 2. The Process of Hot-Dip Galvanizing

**Figure 1:**
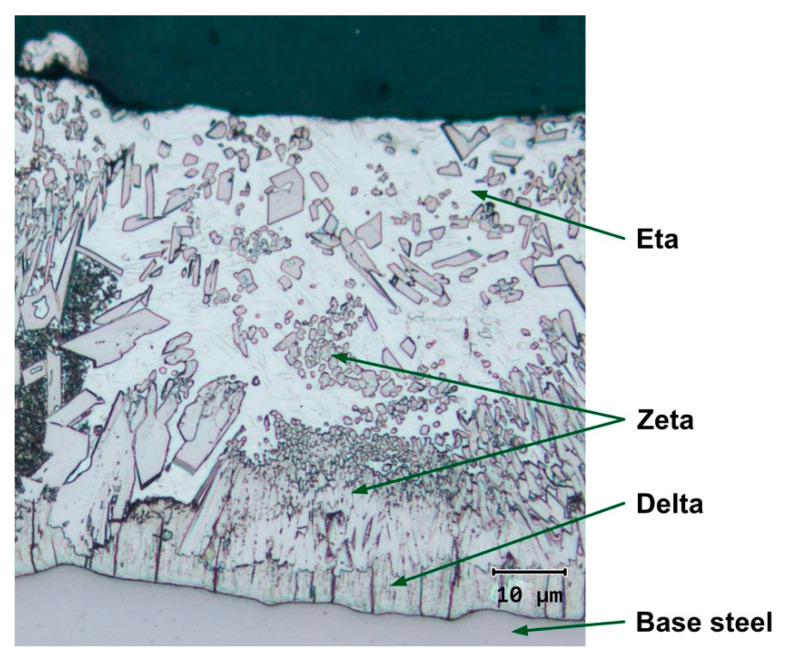
Photomicrograph of a typical hot-dip galvanized steel coating: intermetallic phases of iron (delta, zeta) and zinc (eta).

Hot-dip galvanizing is a metallurgical process where a coating is created on the surface of a steel sheet by mutual reaction of the base material of the product and molten zinc from a bath [18]. The thickness, structure, and quality of the zinc coating are strongly impacted by the composition of the molten zinc and by the condition of the steel surface [19]. Within the metallurgical reaction of iron with molten zinc, intermetallic phases of iron and zinc are gradually created (gamma, delta, zeta). The phases form layers in which the content of iron drops from the zinc–iron interface towards the surface (see Figure 1): The zeta phase contains 5.8 to 6.7 wt.% Fe, the delta phase 7 to 11.5 wt.% Fe, and the gamma phase 21 to 28 wt.% Fe. During the extraction from the bath, a layer of pure Zn (eta phase) with <0.03 wt.% Fe is formed on the surface [20].

Sheet metal and structures are galvanized with almost pure zinc and alloying elements, which are not soluble in the solid eta phase of zinc (Sn, Ni, Pb). These elements form randomly oriented crystals during the crystallization of the surface layer of the zinc.

Hot-dip galvanizing is mostly carried out in baths at temperatures ranging from 445 to 460 °C [6,21]. The metallurgical processes themselves are influenced by the involved alloying elements, mainly Ni, Al, and Sn [22,23]. The optimum amount of nickel in the zinc bath typically ranges from 0.04 to 0.06 wt.%. The content of Ni above 0.06 wt.% leads to the formation of FeZnNi phase particles, the so-called floating dross, which adversely influences the hot-dip galvanizing process.

Nickel (Ni) in the zinc bath reduces the growth speed of zinc coating on the surface of Sandelin steels with the content of Si ranging from 0.03 to 0.12 wt.%. With Sebisty steels (0.12 to 0.22 wt.% of Si) and steels with high Si content (above 0.22 wt.%), Ni does not change the growth kinetics and the growth rate is approximately linear. The presence of higher amounts of Ni in the zinc bath has an unfavorable effect on the creation of hard zinc. On the other hand, if the content of Ni in the bath drops below 0.03%, its influence on the reaction between steel and zinc considerably decreases. Aluminum (Al) is only added in small quantities (0.001 to 0.01%) to increase the luster of the coating. Bismuth (Bi) increases the fluidity of the molten zinc mixture; typically, the content of Bi ranges between 0.1 and 0.2 wt.%. Tin (Sn) gives the zinc layer a characteristic spangle pattern. The content of tin in molten zinc is approximately 1 wt.%. A combination of both Bi and Sn is used to lower the melting temperature of the zinc in the kettle, as the melting temperature of the individual elements is lower than the melting temperature of Zn. Another property of the individual elements is that they are soluble neither in zinc nor in the iron–zinc intermetallic phases. Hence, they form eutectic compounds on the surface of the coating [7,22,23,24,25,26].

As discussed above, the usual temperature of a zinc bath is about 450 °C [7]. The heat introduced within hot-dip galvanizing can adversely impact the mechanical properties of quenched and tempered high-strength steels [27,28]. Steels with a yield strength above 1000 MPa may lose up to 25% of the strength at the temperature of hot-dip galvanization due to the tempering temperature during galvanization being higher than the temperature within manufacturing. The strength of carbon steels, on the other hand, does not change or may slightly increase during hot-dip galvanizing. The elongation does not change but the impact energy mildly decreases. Residual tension after welding decreases by hot-dip galvanizing.

Fatigue strength of the hot-dip galvanized steels is given by the type of steel. In the case of aluminum-killed steel, there is only a relatively small decrease in strength; however, in the case of silicon-killed steel, the decrease can be significant. The cause of these differences is the differing composition of the intermetallic phases of iron and zinc. As a result of the fatigue stress, fractures in the coating are created. These fractures subsequently initiate the formation of cracks in the surface of the steel. As long as the zinc coating on the steel surface remains intact, the fatigue strength is not impacted by the coating and the decrease in fatigue strength caused by hot-dip galvanizing is significantly smaller than the decrease otherwise caused by the corrosion attack [24].

Furthermore, hot-dip galvanizing does not cause an increase in hydrogen embrittlement, since the hydrogen absorbed during pickling by hydrochloric acid is subsequently released by the heat from the process. Intergranular embrittlement, which can occur in certain cases with hot-dip galvanizing, is caused by the penetration of zinc into the borders of the steel grains. This phenomenon occurs due to increased tension in the structural element. It is therefore recommended to anneal the steel at a temperature higher than the temperature in molten zinc, i.e., above 460 °C [29,30].

## 3. Materials and Methods

### 3.1. Properties of the Selected Steels

Three groups of high-strength steels produced by SSAB Oxelösund were used for the experimental verification of the effect of hot-dip galvanizing on the mechanical properties of steels: DOMEX, HARDOX, and ARMOX. DOMEX steels in classes 460, 550, and 700 are structural high-strength steels with a high level of nominal yield strength (460 to 700 MPa) and tensile strength (520 to 750 MPa) [31,32,33]. HARDOX steels in classes 500 and 600 are high-strength and highly abrasion-resistant steels with a high level of nominal hardness (500 and 600 HBW) and toughness [8,9]. The armor steel ARMOX is in class 500 and has a high level of nominal hardness (500 HBW), toughness, and strength (tensile strength up to 1400 MPa) [10].

According to the recommendations of the manufacturer, HARDOX and ARMOX steels are not intended for further heat treatment, which may also include a hot-dip galvanizing process [8,9,10]. The performed experimental analyses aimed at the verification of the mechanical properties (strength and hardness) of the materials after the hot-dip galvanizing. Quantification of the changes was also performed.

#### 3.1.1. DOMEX 460, 550, and 700

DOMEX is a series of high-strength hot-rolled steels, which are intended mainly for cold-forming. Their material characteristics are summarized in Table 2 and Table 3 [31,32,33]. They are characterized by high strength, excellent formability, and good weldability. They are fabricated by the process of thermomechanical rolling with quenching and tempering. The parameters of the process are optimized to achieve the desired levels of strength. Due to their low carbon and high manganese content, they are suitable for welding. This type of steel is currently used mainly in heavy machinery in the engineering and automobile industry (heavy-duty parts).

The properties of the materials, particularly their mechanical characteristics, imply the suitability of the materials for use in the load-bearing structures of buildings and technical structures, and the construction of steel and steel–concrete bridges. In other words, it is employed in situations where it is necessary to provide high strength, rigidity, toughness, and mechanical resistance, and to achieve low weight at the same time.

#### 3.1.2. HARDOX 500, 600

Table 4 and Table 5 summarize the material characteristics of HARDOX steels [8,9]. These are high-strength (up to 1200 MPa), quenched, and slightly tempered abrasion-resistant steels with high hardness (up to 600 HB). HARDOX steels combine the characteristics of structural steels and abrasion-resistant steels. They have high resistance, toughness, and hardness in the entire material width, i.e., not only on the surface. These properties provide a long lifetime in the most adverse conditions. The steels are well workable by cutting and bending, and well weldable.

The steels are used mainly in applications with requirements of a very high lifespan and resistance to abrasive conditions. HARDOX steel plates are used in the mining industry: transport and processing of mineral raw materials.

#### 3.1.3. ARMOX 500

The highest yield strength attained by high-strength steel ARMOX 500 is 1300 MPa. It has low elongation, and high abrasion and ballistic resistance. The high strength is achieved by heat processing and alloying: Ni–1.8%, Cr–1.0%, and C–0.32% (see Table 4 and Table 5 [10]). It is commonly used in military applications and the special automobile and construction industry. It is mainly welded using austenitic electrodes at locations where the base material is under minimum stress.

### 3.2. Preparation of the Specimens

The steel samples were cut using an ESAB LPH 50 air plasma cutter and blasted with brown corundum No. 22 with a grain size of 0.6–1 mm. The basic dimensions of the cross-sections of test specimens were 6 × 25 mm for DOMEX 460, 550, and 700; 6 × 20 mm for HARDOX 500 and 700; and 8 × 20 mm for ARMOX 500. The exact dimensions of the cross-section were measured for each test specimen individually.

The hot-dip galvanizing of high-strength steel was performed in the standard way at the company Wiegel CZ with the temperature of the process set to 455 °C. After cutting, the specimens underwent alkaline degreasing and pickling with the use of 12% chloric acid. Subsequently, the flux was applied (zinc chloride and ammonium chloride). Hot-dip galvanizing was performed with an immersion period of 3 min. The zinc mixture contained Ni, Al, and Bi. The content of the elements is the know-how of the company.

**Figure 2:**
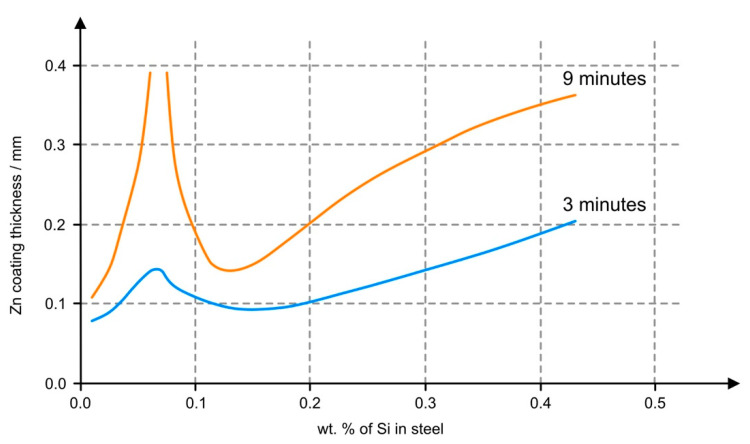
Relationship between the silicon content in the steel and the thickness of the zinc coating [35].

Figure 2 shows the dependence of zinc coating layer growth speed on the Si content in the steel. The steels used in this study could be divided accordingly into two groups. The first group included DOMEX 460, 550, and 700. The DOMEX had a total Si content of up to 0.1 wt.%, and a P content of up to 0.025 wt.%. The steels of the second group, HARDOX 500 and 600, and ARMOX 500, had up to 0.7% Si (typically 0.15 to 0.28 wt.%). The coating structure contained long, sharp crystals of intermetallic phases [34].

The thickness of the zinc layers was verified using coating thickness gauge PosiTector 6000-FNS3 (see Table 6). This device is capable of performing measurements of thickness in the range of 1 to 2000 µm. It is intended for the measurement of non-ferrous coatings on ferromagnetic and non-ferromagnetic materials. The thickness of ferromagnetic layers is measured based on magnetic induction. With non-ferrous materials, the measurement of the eddy current is employed.

**Figure 3:**
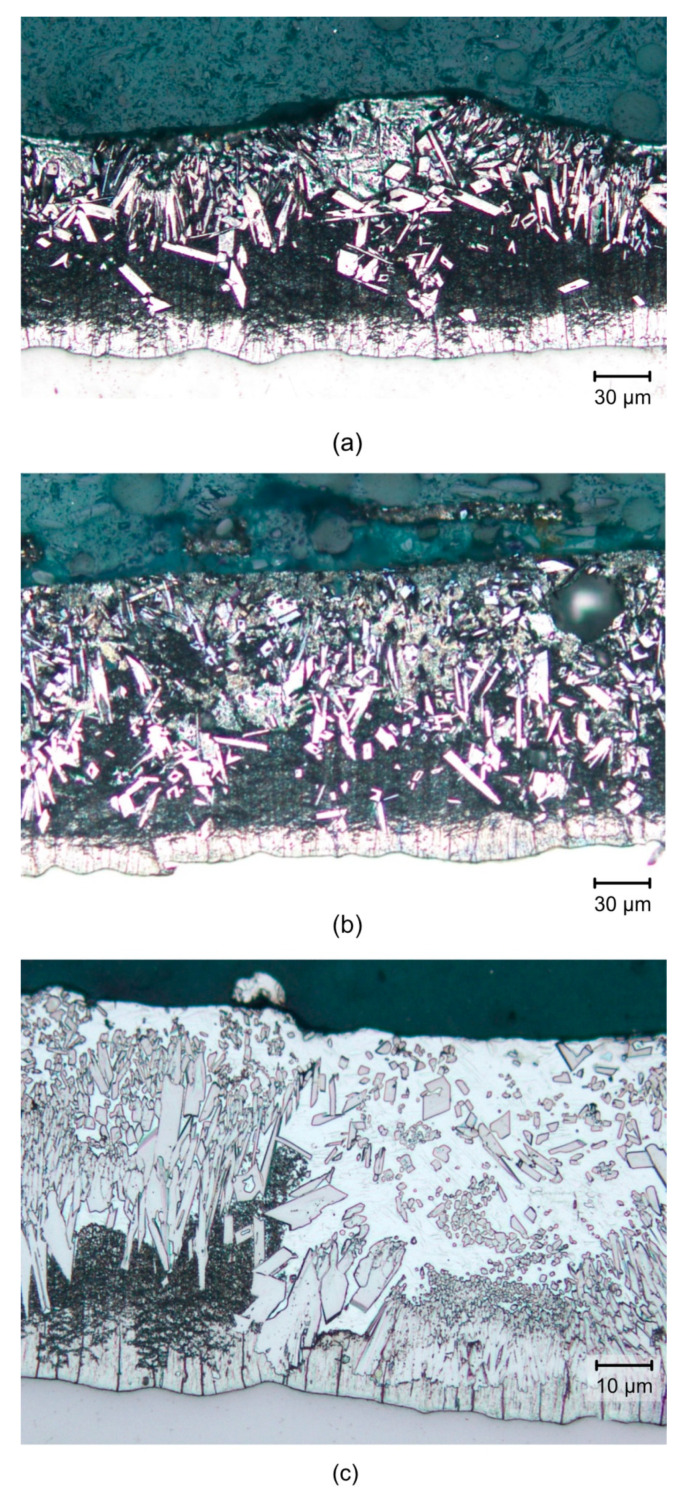
Photomicrographs of the structure of the coating on the surface of the tested high-strength steel specimens, formed by the process of hot-dip galvanizing: (**a**) HARDOX 600, (**b**) ARMOX 500, (**c**) DOMEX 700.

Figure 3a shows the microphotograph of the zinc coating on the surface of a HARDOX 600 specimen. The thickness of the zinc coating was about 120 μm. It was formed by delta phase crystals in the thickness of 30 μm, which adhered to the base material, and by large randomly oriented zeta-phase crystals reaching the surface of the coating. The eta phase—pure zinc—only formed a thin top layer of the coating with HARDOX 600.

Figure 3b shows the microphotograph of the zinc coating on the surface of the ARMOX 500 steel specimen. The coating thickness reached approximately 120 μm, and the coating consisted of a layer of delta phase with a thickness of approximately 20 μm, randomly oriented crystals of the zeta phase, and the eta phase.

Figure 3c shows the microphotograph of the zinc coating on the surface of the DOMEX 700 steel specimen. The thickness of the coating was approximately 80 μm. The coating consisted of a layer of delta phase with a thickness of approximately 10 μm, a zeta phase, and an eta phase containing randomly oriented zeta-phase crystals.

The structure and thickness of the zinc coating corresponded to the typical chemical composition of these steels, particularly to the Si content (see Figure 2). HARDOX steels contain 0.15 to 0.28 wt.% of Si, ARMOX steels contain 0.20 to 0.26 wt.% of Si, and DOMEX steels contain less than 0.03% of Si.

### 3.3. Strength Measurements

The determination of the yield strength *R_p_*_0.2_ and the tensile strength *R_m_* was carried out using tearing instrument ZD-40 and the evaluation was performed using program M-Test 1.75. The instrument is equipped with an incremental distance sensor for the position of the crossbar with a resolution of 0.01 mm, and a force sensor with driving unit EDC 60.

The static tension tests were performed according to the standard document EN ISO 6892-1 [36], and the used strain rate was 10 MPa/s. The size and shape of the test specimens were prepared according to Appendix D of the normative document.

### 3.4. Surface Hardness Measurements

The hardness of a material is one of the most basic mechanical properties. The hardness measurement was performed by the method by Vickers with a load of 98.07 N, as recommended by EN ISO 6507-1: 2018 [37], and the holding time was 15 s. It was measured with the use of the Zwick 3212 instrument. The hardness HV10 was measured on the surface of the test specimens (after removing the zinc coating and sample polishing). For the specimens with zinc coating, the layer of zinc was removed by grinding before the measurement. The surface was metallographically prepared prior to measurement: The cutting was performed with the use of laboratory saw Struers Labotom 5, and the surface was subsequently processed with the use of sandpapers with grit sizes 240 to 1200 and polished with the use of DP-paste 15 μm. Finally, the surface was etched with the use of Nital Etchant (10%). Five measurements were performed with each sample.

## 4. Results

### 4.1. Tensile Tests

The extent of the change in the mechanical properties of selected high-strength steels, resulting from the use of hot-dip galvanizing, was experimentally measured. In the experiments, the yield strength *R_p_*_0.2_, the tensile strength *R_m_*, and the hardness of the material were determined.

**Figure 4:**
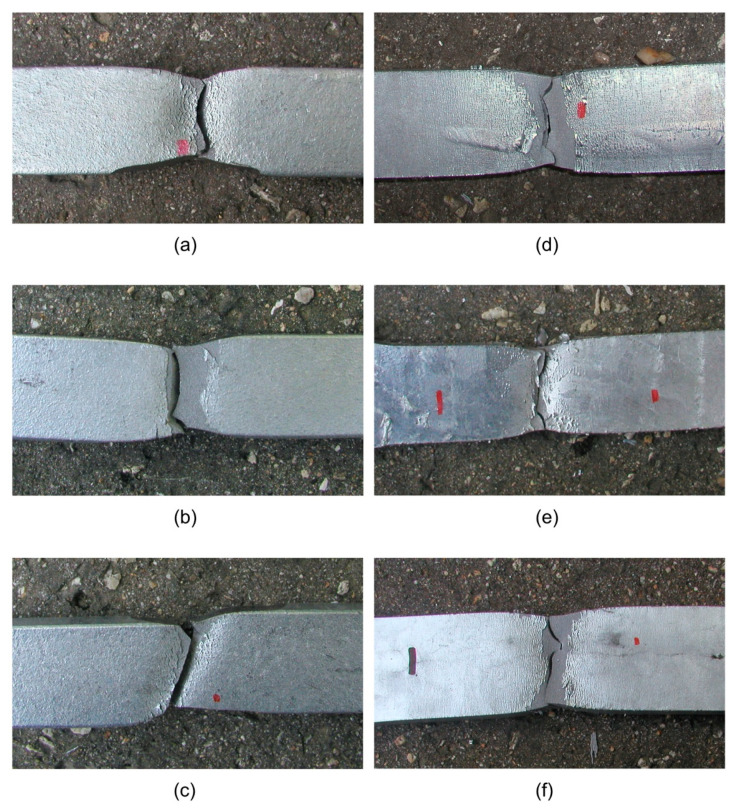
Tested hot-dip-galvanized specimens after the tensile tests were performed: (**a**) DOMEX 460, (**b**) DOMEX 550, (**c**) DOMEX 700, (**d**) HARDOX 500, (**e**) HARDOX 600, and (**f**) ARMOX 500.

Figure 4 shows selected samples made of DOMEX, HARDOX, and ARMOX steels after the tensile tests were performed. In the case of the DOMEX steels, which are tougher and have higher elongation, the deformation of the steel was higher and thus, the peeling of the zinc layer was greater than in the case of HARDOX and ARMOX steels. The fracturing mode was also different in these steels: Ductile fractures appeared in the case of DOMEX-brand steels; the steels with a higher yield strength (i.e., the HARDOX and ARMOX brands) showed, on the other hand, cleavage fracture.

**Figure 5:**
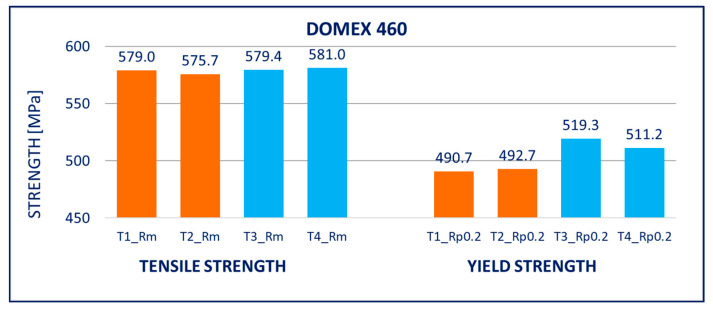
Tensile tests of specimens from DOMEX 460 steel. Description of specimens: T1 and T2 are groups of specimens without zinc coating; T3 and T4 are groups of specimens after hot-dip galvanization. *R_m_* is tensile strength, *R_p_*_0.2_ is yield strength.

**Figure 6:**
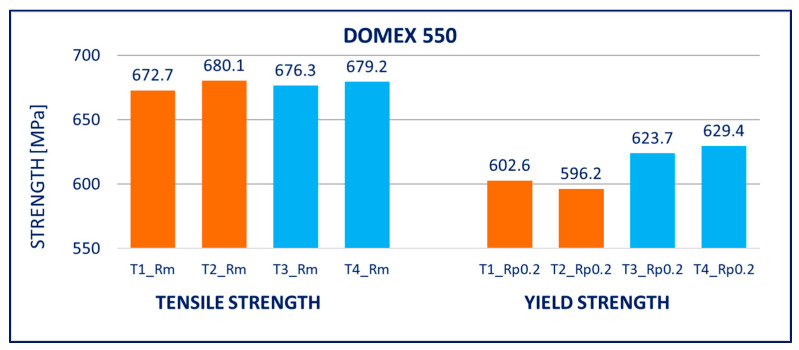
Tensile tests of specimens from DOMEX 550 steel. Description of specimens: T1 and T2 are groups of specimens without zinc coating; T3 and T4 are groups of specimens after hot-dip galvanization. *R_m_* is tensile strength, *R_p_*_0.2_ is yield strength.

**Figure 7:**
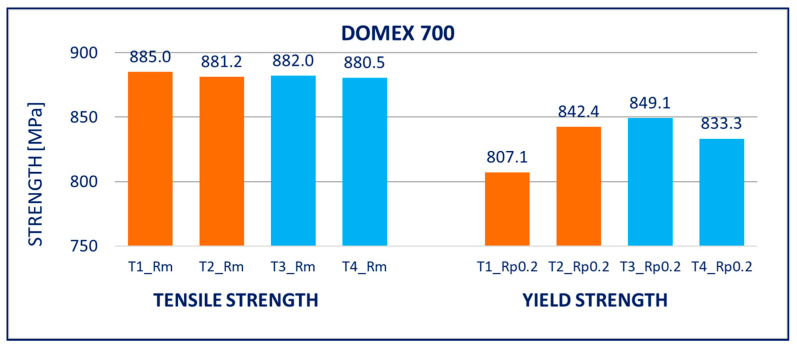
Tensile tests of specimens from DOMEX 700 steel. Description of specimens: T1 and T2 are groups of specimens without zinc coating; T3 and T4 are groups of specimens after hot-dip galvanization. *R_m_* is tensile strength, *R_p_*_0.2_ is yield strength.

**Figure 8:**
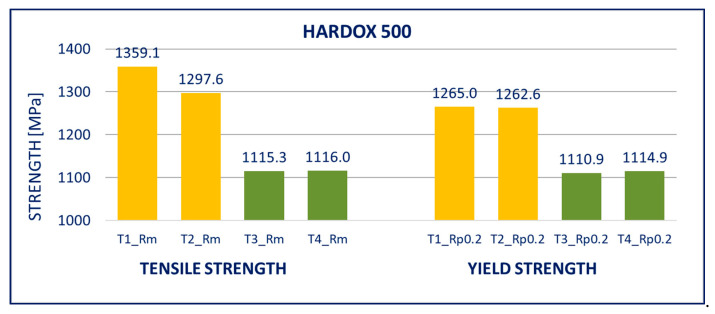
Tensile tests of specimens from HARDOX 500 steel. Description of specimens: T1 and T2 are groups of specimens without zinc coating; T3 and T4 are groups of specimens after hot-dip galvanization. *R_m_* is tensile strength, *R_p_*_0.2_ is yield strength.

**Figure 9:**
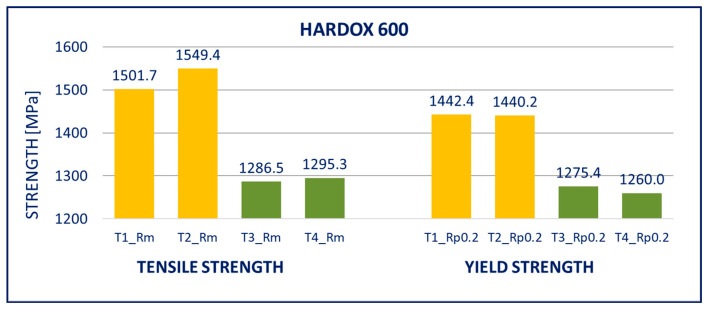
Tensile tests of specimens from HARDOX 600 steel. Description of specimens: T1 and T2 are groups of specimens without zinc coating; T3 and T4 are groups of specimens after hot-dip galvanization. *R_m_* is tensile strength, *R_p_*_0.2_ is yield strength.

**Figure 10:**
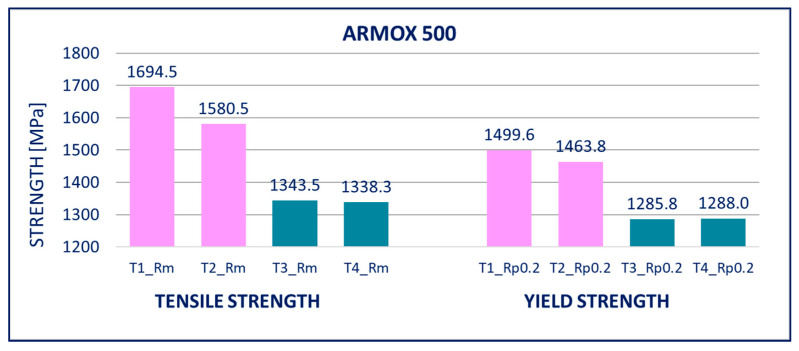
Tensile tests of specimens from ARMOX 500 steel. Description of specimens: T1 and T2 are groups of specimens without zinc coating; T3 and T4 are groups of specimens after hot-dip galvanization. *R_m_* is tensile strength, *R_p_*_0.2_ is yield strength.

The results of tensile tests of the specimens of DOMEX 460, DOMEX 550, DOMEX 700, HARDOX 500, HARDOX 600, and ARMOX 500 materials with and without hot-dip galvanizing are provided in Figure 5, Figure 6, Figure 7, Figure 8, Figure 9 and Figure 10.

### 4.2. Hardness

The hardness of the hot-dip galvanized steels was significantly lower in comparison to the non-galvanized steels. The decrease was observed with all tested materials, i.e., with DOMEX, HARDOX, and ARMOX. However, a higher decrease occurred with the steels with higher yield strength *R_p_*_0.2_, i.e., HARDOX and ARMOX. This effect can be explained by the decrease in carbon content at the surface, discussed in Section 5.

**Figure 11:**
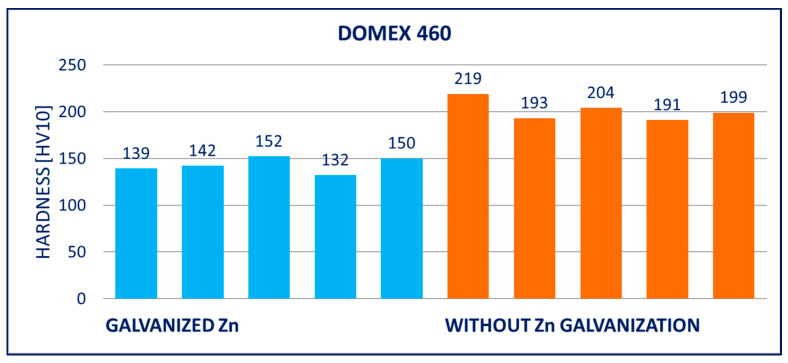
Results of hardness HV10 of specimens of DOMEX 460 steel.

**Figure 12:**
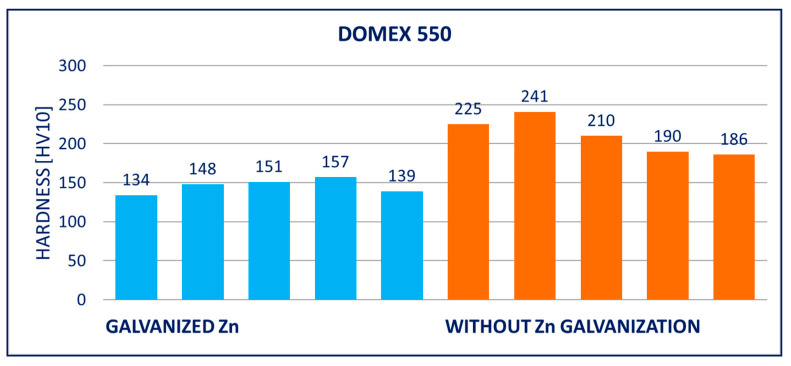
Results of hardness HV10 of specimens of DOMEX 450 steel.

**Figure 13:**
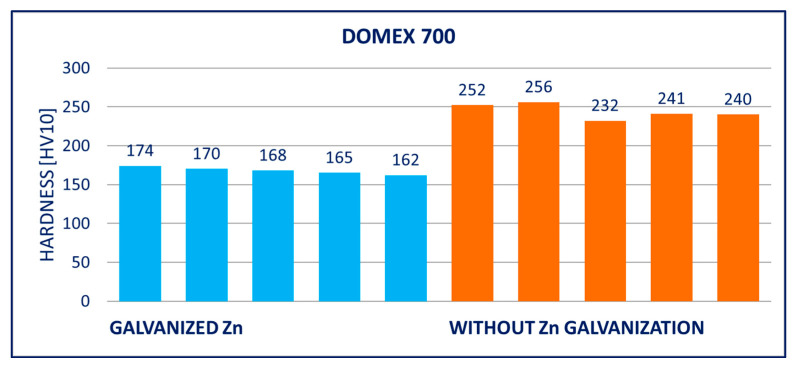
Results of hardness HV10 of specimens of DOMEX 700 steel.

**Figure 14:**
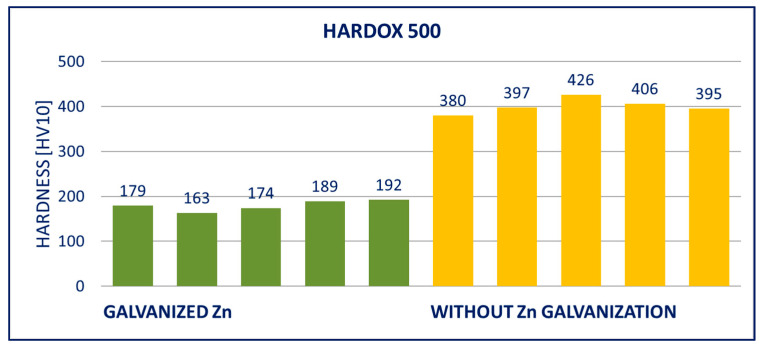
Results of hardness HV10 of specimens of HARDOX 500 steel.

**Figure 15:**
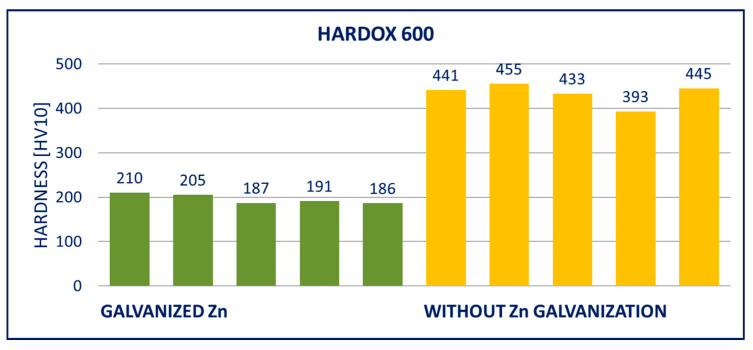
Results of hardness HV10 of specimens of HARDOX 600 steel.

**Figure 16:**
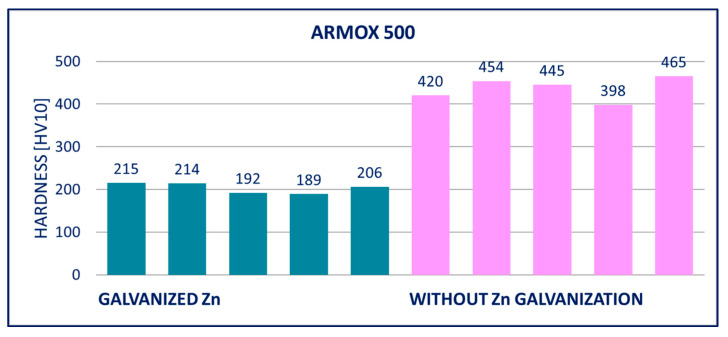
Results of hardness HV10 of specimens of ARMOX 500 steel.

The results of hardness tests of the specimens (measured on the surface of the specimens after removing the zinc coatings) are provided in Figure 11, Figure 12, Figure 13, Figure 14, Figure 15 and Figure 16; hardness HV10 was measured.

## 5. Discussion

**Figure 17:**
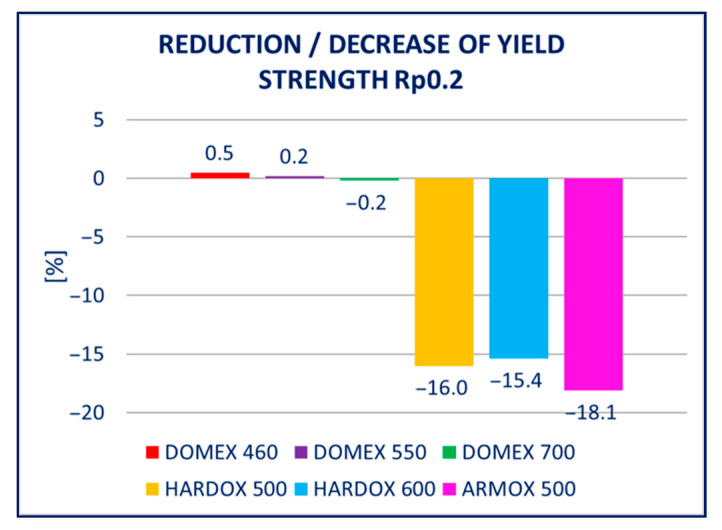
Summary of the results for the yield strength tests presented in the Results section: relative change in *R_p_*_0.2_ for the different types of steel.

**Figure 18:**
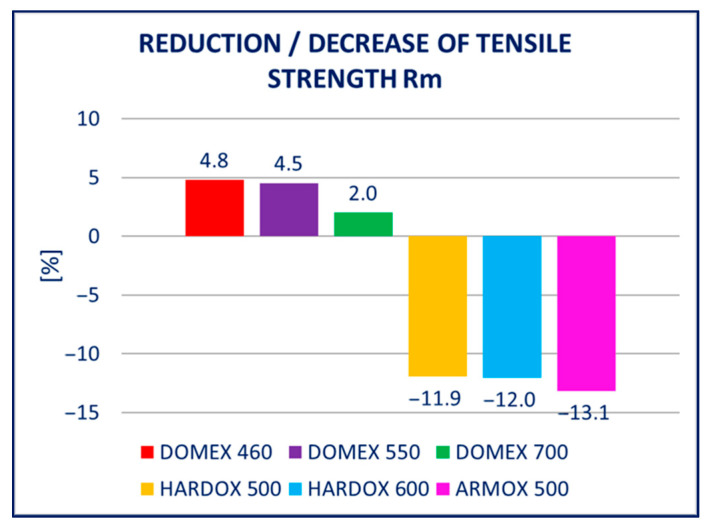
Summary of the results for tensile strength tests presented in the Results section: relative change in *R_m_* for the different types of steel.

**Figure 19:**
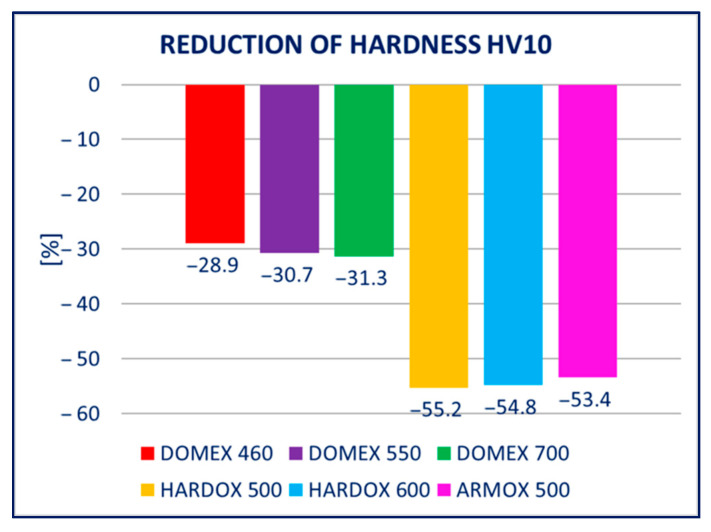
Summary of the results for hardness tests presented in the Results section: relative change in hardness for the different types of steels.

The unfavorable influence of hot-dip galvanizing (a form of heat process) on the yield and tensile strength is a known phenomenon. Several studies devoted to the phenomenon were published, e.g., [27,28]. Overall, the studies dealt with the influence of hot-dip galvanization of the steels with a tensile strength below 700 MPa. Our study included the different types of high-strength steels with tensile strength ranging from 600 to 1800 MPa. The study aimed to verify and quantify the influence of hot-dip galvanizing on the yield and tensile strength (summarized in Figure 17 and Figure 18), but also to evaluate the impact on the hardness of the material (summarized in Figure 19).

DOMEX 460: After hot-dip galvanizing, the mechanical properties of the steel improved. The yield strength *R_p_*_0.2_ was increased by 20 to 30 MPa and the tensile strength *R_m_* also increased slightly. The elongation *A* did not change. The experiment did not show any negative impact of hot-dip galvanization on the mechanical properties of this steel; instead, slight improvements were recorded.

DOMEX 550: The mechanical properties slightly changed after hot-dip galvanizing. The yield strength *R_p_*_0.2_ was increased by 20 to 30 MPa and the tensile strength *R_m_* remained unchanged; however, the elongation *A* increased by 14%. The experiments showed that the hot-dip galvanization of this steel does not have a negative influence on its strength.

DOMEX 700: The mechanical properties remained almost unchanged after hot-dip galvanization. The yield strength *R_p_*_0.2_, the tensile strength *R_m_*, and the elongation *A* remained constant and corresponded to the initial values, i.e., the values before galvanizing.

HARDOX 500: The mechanical properties of this steel were significantly worse after hot-dip galvanizing. The yield strength *R_p_*_0.2_, as well as the tensile strength *R_m_*, decreased by almost 150 MPa because of the galvanization process. Elongation *A* was not significantly changed by galvanizing.

HARDOX 600: The mechanical properties of the steel deteriorated very significantly by the process of hot-dip galvanizing. It affected mainly the yield strength *R_p_*_0.2_ and tensile strength *R_m_*. Elongation *A* remained unchanged. The yield strength *R_p_*_0.2_ decreased by almost 150 MPa and the tensile strength *R_m_* by more than 250 MPa.

ARMOX 500: After hot-dip galvanizing, significant weakening of the mechanical properties was observed. The yield strength *R_p_*_0.2_ and the ultimate strength *R_m_* were mainly impacted, whereas elongation *A* only increased slightly. The yield strength *R_p_*_0.2_ decreased by approximately 300 MPa and the tensile strength *R_m_* by more than 400 MPa. Out of all the tested steels, hot-dip galvanization had the most negative impact on ARMOX 500.

Within the manufacturing process, HARDOX and ARMOX steels are tempered to a temperature of approximately 200 °C. Due to the low tempering temperature, the steels consist mainly of tetragonal martensite, and the yield strength ranges above 1200 MPa. Decomposition of the tetragonal martensite to cubic martensite with lower carbon content and transition iron carbide (ε-carbide) with a close-packed hexagonal structure is the key process occurring during tempering of the steels. At temperatures between 200 and 300 °C, the residual austenite decomposes, and lower bainite is formed. This structure is similar to the structure of martensite tempered to the same temperature. When the steel is heated above 300 °C, the low-carbon martensite decomposes to ferrite, and simultaneously, cementite is formed. The amount of precipitate of cementite gradually increases as particles of ε-carbide decay. Simultaneously, the C content in the matrix decreases due to the formation of stable carbide Fe_3_C (cementite). These changes lead to decreased strength (Figure 8, Figure 9 and Figure 10) and hardness (Figure 14, Figure 15 and Figure 16) and increased plasticity and toughness. The conclusions are supported by the different hardness measured on the surface of the specimens (after removal of the zinc coating) and in the core (discussed in the 10th paragraph of this section). The dependence of the decrease of hardness on the temperature is discussed in the available literature [38,39].

The structural phases are stable with the DOMEX steels, which are tempered to 550 °C during the manufacturing process. Hence, the changes in the mechanical properties resulting from hot-dip galvanizing are not significant. On the other hand, the DOMEX steels are microalloyed with Ti, Nb, and V. As a result of the heat process, the formation of carbides, nitrides, and carbonitrides may occur. The presence of these phases explains the slight increase in the tensile mechanical properties.

Softening of the surface parts occurs as a result of the process of hot-dip galvanization. A light layer below the grinded zinc coating was observed in photomicrographs. There was a decreased C content in this layer, which negatively impacted the hardness. The extent of this effect can be illustrated in the example of the DOMEX 700 and HARDOX 500 steels. The hardness was measured with the method by Vickers HV10. The hardness HV10 measured in the softened zone of a test specimen made of DOMEX 700 ranged from 185 to 208. The hardness HV10 measured at the core of the specimen was in the interval of 256 to 268. For comparison, the hardness HV10 of the non-galvanized specimen ranged from 256 to 272. The hardness HV10 measured in the softened zone of a test specimen made of HARDOX 500 and at the core of the specimen ranged from 202 to 230 and from 316 to 339, respectively. The hardness HV10 of the non-galvanized specimen ranged from 378 to 415.

Very recently, a study devoted to hot-dip galvanizing of thin-walled steel tubes made of ultra-high-strength steels was published [40]. The studied steels contained 0.23 wt.% of C, 0.8 wt.% of Si, 1.7 wt.% of Mn, 1.5 wt.% of Cr, 1.0 wt.% of Ni, 0.5 wt.% of Mo, and 0.005 wt.% of B. The authors reported a decrease in the strength of the steels from 1373 MPa (before galvanization) to 1100 MPa (after galvanization). Similar results were obtained by Gunalan and Mahendran [41], who reported a decrease in strength of 17%, and by Azhari et al. [42], who reported a decrease in strength of 14%. The published structures of the zinc coating show high similarity with our results: the relatively low thickness of the delta phase and large randomly oriented crystals of the zeta phase.

The performed experiments show the negative influence of hot-dip galvanizing on the hardness of the material. All types of steel suffered from a significant decrease in hardness, particularly steels with higher yield strength. The smallest decrease in hardness resulting from the galvanization process was measured for DOMEX steels. The decrease ranged from 60 HV (DOMEX 460) to 80 HV (DOMEX 700). With HARDOX steels, the observed decrease in hardness was 220 HV and 240 HV for HARDOX 500 and 600, respectively. With ARMOX 500 steel, a decrease in hardness of 230 HV resulting from hot-dip galvanizing was observed.

## 6. Conclusions

Experimental research has confirmed that hot-dip galvanizing causes a significant deterioration of the mechanical properties of selected types of high-strength steels.

The key advantage of DOMEX-type high-strength steels is the high strength of the material (yield and tensile strength). Only a slight change in the mechanical properties after hot-dip galvanization was observed with steels with a characteristic value of yield strength of 460 MPa, 550 MPa, and 700 MPa. There was a slight increase in the yield strength; however, the ultimate strength remained almost unchanged by the process. Plastic properties, namely, the elongation, also remained unchanged for most steel classes. Hot-dip galvanizing can be recommended as an anti-corrosive protection for this type of steel without any major drawback.

HARDOX and ARMOX steels are characteristic by their high hardness together with the high strength of the materials. They have high yield strength (min. 1200 MPa) and tensile strength (1350 to 1750 MPa). With this type of steel, the change in yield strength and tensile strength resulting from the hot-dip galvanization was tremendous. The yield strength of these steels dropped by up to 150 MPa, and the ultimate strength fell by more than 200 MPa. At the same time, the elongation increased due to the drop in strength and hardness.

The influence of hot-dip galvanization on the hardness of high-strength steels is also significant. The weakening of strength and hardness occurs due to the additional tempering of the martensitic structure. All types of steel suffered from a significant decrease in hardness, particularly the steels with higher yield and tensile strength. In the case of the DOMEX-type steels, the measured decrease was mostly around 70 HV. The decrease in hardness of approximately 60 HV on average was observed for steels with a yield strength of up to 600 MPa, approximately 75 HV for the steels with a yield strength of 700 MPa. In all cases, the decrease in hardness was approximately 30%. In the case of the ARMOX and HARDOX steel types, the hardness decreased by a maximum of 220 to 240 HV due to the hot-dip galvanization, which was approximately 55%.

The results imply that only steels with lower strength are suitable for hot-dip galvanization. The upper level of the yield-strength limit was estimated as *R_p_*_0.2_ = 700 MPa. Steels with a higher yield-strength level and high hardness, e.g., HARDOX and ARMOX steels, are tempered to only 200 °C during production. The change in the mechanical properties is due to the influence of the galvanizing temperature (usually ranges from 445 to 460 °C).

Anti-corrosive protection of this type of steel with the use of hot-dip galvanizing can be recommended only for high-strength steels with a yield strength below 700 MPa and high-strength steels for which the extent of the decrease in the mechanical properties is known.

## Figures and Tables

**Table 1:** The corrosion rate of steel and zinc with respect to corrosivity categories (EN ISO 9223) [4].

Category	Corrosion Risk	Corrosion Rate *R_corr_* for the First Year of Exposure			
		Low-Carbon Steel		Zinc	
		Mass Loss (G/M^2^)	Thickness Loss (µm)	Mass Loss (G/M^2^)	Thickness Loss (µm)
C1	Very low	≤10	≤1.3	≤0.7	≤0.1
C2	Low	>10 to ≤200	>1.3 to ≤25	>0.7 to ≤5	>0.1 to ≤0.7
C3	Medium	>200 to ≤400	>25 to ≤50	>5 to ≤15	>0.7 to ≤2.1
C4	High	>400 to ≤650	>50 to ≤80	>15 to ≤30	>2.1 to ≤4.2
C5	Very high	>650 to ≤1500	>80 to ≤200	>30 to ≤60	>4.2 to ≤8.4
CX	Extreme	>1500 to ≤5500	>200 to ≤700	>60 to ≤80	>8.4 to ≤25

**Table 2:** Metal analysis of the SSAB DOMEX steels used. The content of the elements is provided in wt.% (SSAB datasheets) [31,32,33].

Material	C	Si	Mn	P	S	Al	Nb	Ti	V
DOMEX 460	max. 0.1	max. 0.1	max. 1.5	max. 0.025	max. 0.01	min. 0.015	max. 0.09	max. 0.15	max. 0.2
DOMEX 550	max. 0.12	max. 0.1	max. 1.8	max. 0.025	max. 0.01	min. 0.015	max. 0.08	max. 0.15	max. 0.2
DOMEX 700	max. 0.12	max. 0.1	max. 2.1	max. 0.025	max. 0.01	min. 0.015	max. 0.09	max. 0.15	max. 0.2

**Table 3:** Basic mechanical properties of DOMEX 460 steels (SSAB datasheets) [31,32,33].

Material	Yield Strength *R_p_*_0.2_	Tensile Strength *R_m_*	Elongation *A*
DOMEX 460	min. 460 MPa	520–670 MPa	min. 15%
DOMEX 550	min. 550 MPa	600–760 MPa	min. 14%
DOMEX 700	min. 700 MPa	750–950 MPa	min. 12%

**Table 4:** Metal analysis of the SSAB HARDOX and ARMOX steels used. The content of the elements is provided in wt.% (SSAB datasheets) [8,9,10].

Material	C	Si	Mn	P	S	Al	Nb	Ti	V
HARDOX 500	max. 0.27	max. 0.7	max. 1.6	max. 0.025	max. 0.01	max. 0.25	max. 0.25	max. 0.004	max. 1.0
HARDOX 600	max. 0.27	max. 0.7	max. 1.6	max. 0.025	max. 0.01	max. 0.25	max. 0.25	max. 0.004	max. 1.0
ARMOX 500	max. 0.32	max. 0.4	max. 1.2	max. 0.015	max. 0.01	min. 1.8	max. 0.7	max. 0.005	max. 1.0

**Table 5:** Basic mechanical properties of HARDOX 500 steel (SSAB datasheets) [8,9,10].

Material	Yield Strength *R_p_*_0.2_	Tensile Strength *R_m_*	Typical Hardness HBW	Elongation *A*
HARDOX 500	min. 1200 MPa	1350 MPa	450–540	min. 5%
HARDOX 600	min. 1200 MPa	1400 MPa	550–640	min. 5%
ARMOX 500	min. 1250 MPa	1450–1750 MPa	515	min. 5%

**Table 6:** The measured thickness of the zinc coating.

Material	Specimens	Mean Thickness of the Zinc Coating (µm)
DOMEX 460	D4-1	80.0 ± 5.1
	D4-2	68.3 ± 2.3
DOMEX 550	D5-1	52.5 ± 1.7
	D5-2	65.0 ± 6.1
DOMEX 700	D7-1	78.4 ± 4.8
	D7-2	78.6 ± 3.4
HARDOX 500	H5-1	144.0 ± 9.9
	H5-2	139.2 ± 9.8
HARDOX 600	H6-1	134.4 ± 4.1
	H6-2	120.4 ± 6.0
ARMOX 500	A1-1	126.4 ± 11.6
	A1-2	130.4 ± 9.8
